# Impact of frailty severity and severe pain on cognitive function for community-dwelling older adults with arthritis: a cross-sectional study in Korea

**DOI:** 10.1038/s41598-024-53431-3

**Published:** 2024-02-04

**Authors:** Wonhee Baek, Yujin Suh, Yoonjung Ji

**Affiliations:** 1 College of Nursing, Gyeongsang National University, Jinju, Gyeongnam South Korea; 2 Healthcare Sciences and the Human Ecology Research Institute, Department of Nursing, Healthcare Sciences and the Human Ecology, Dong-eui University, Busan, South Korea; 3 Brain Korea 21 FOUR Project, Mo-Im Kim Nursing Research Institute, College of Nursing, Yonsei University, 50-1 Yonsei-ro, Seodaemun-gu, Seoul, 03722 South Korea

**Keywords:** Health care, Geriatrics, Public health, Quality of life

## Abstract

Pain is a major symptom of arthritis in older adults, often leading to frailty and cognitive decline. However, few studies have investigated the relationship among pain, frailty, and cognitive function in older adults with arthritis. This study aimed to investigate the factors influencing cognitive function and the impact of frailty severity and pain on cognitive function in older adults with arthritis using a Korean population-based dataset. This cross-sectional descriptive study involved the secondary data of 1089 participants from the seventh and eighth waves of the Korean Longitudinal Study on Aging. We examined general characteristics, health behaviors, health conditions (including severe pain and frailty), and cognitive function. Participants were categorized based on the presence or absence of pain severity and frailty status as follows: robust, only severe pain, only prefrail, prefrail with severe pain, only frail, and frail with severe pain. Multiple linear regression analysis was performed to establish correlations between groups and cognitive function. The only-prefrail group was the largest (19.7%) among participants experiencing either pain or frailty. Advanced age, sex, level of education, and visual and hearing impairments were significantly associated with cognitive function. Compared to the robust group, only prefrail (*β* = -1.54, confidence interval [CI] = − 2.33; − 0.76), prefrail with severe pain (*β* = − 2.69, CI = − 3.52; − 1.87), only frail (*β* = − 4.02, CI = − 5.08; − 2.97), and frail with severe pain (*β* = − 5.03, CI = − 5.99; − 4.08) groups were associated with lower Mini-Mental State Examination scores. The study confirmed that severe pain alone does not significantly impact cognitive function in older adults with arthritis. To prevent cognitive decline in this group, assessment of both pain and frailty severity is essential to predict high-risk groups and provide appropriate interventions, such as transfer to hospitals or primary clinics according to the severity of pain and frailty.

## Introduction

By 2050, the population of older adults is expected to double, with an estimated 425 million people aged 80 years and older^1^. Concomitantly, the burden of degenerative diseases due to aging is anticipated to increase. Osteoarthritis (OA), one of the most common degenerative diseases in older adults, affects an estimated 10.5% of the US population^2^, whereas rheumatoid arthritis (RA), a chronic autoimmune disease, has a prevalence of 0.5–1.0% in Europe and North America^3^. The risk of both conditions increases with age^2^. Patients with arthritis experience symptoms such as pain, joint impairment, and fatigue along with associated disease burdens such as reduced quality of life and work productivity^4^.

Cognitive decline is a notable symptom of aging, with previous studies reporting its relevance in patients with arthritis^5^, particularly affecting attention, memory, and verbal function. Cognitive decline was reported in 67% of patients with RA in the UK^6^, and the incidence of dementia in patients with OA was twice that of non-OA in Taiwan^7^. While depression, chronic pain, drug-related effects, functional disabilities, and inflammation have been proposed as potential explanations for the cognitive decline observed in patients with arthritis, the precise mechanism underlying this decline remains unclear^8^. Inflammation has been hypothesized to be the biological mechanism that links arthritis and cognitive impairment. In arthritis, peripheral inflammation and cytokine release can induce changes in brain metabolism. This inflammation can be exacerbated by the production of amyloid peptides, which contribute to the formation of brain lesions in Alzheimer’s disease^9,10^. However, several studies have found that older adults with arthritis experience cognitive decline at a similar rate to those without arthritis, suggesting that arthritis may not accelerate the development of cognitive impairment^11,12^. Given these contradictory findings, it is beneficial for healthcare providers to assess the cognitive function of patients with arthritis by identifying the degree of cognitive decline based on representative population data and relevant influencing factors.

Chronic pain, one of the most prevalent and burdensome symptoms of arthritis in older adults^4^, can be alleviated through appropriate approaches and may be a modifiable factor in improving or reversing geriatric conditions^13–16^. Unresolved pain often has detrimental consequences, including severe disability due to reduced mobility, falls, depression, anxiety, disturbed sleep, isolation, and sarcopenia^17,18^. In a randomized controlled trial that examined pain and cognitive decline in healthy community-dwelling older adults, pain was confirmed as a contributing factor to the acceleration of cognitive decline^19,20^. In previous studies, pain has been strongly associated with cognitive decline in arthritis patients^5^.

Frailty refers to a state in which an individual's vulnerability increases due to a decrease in the ability to maintain homeostatic balance. The phenomenon of increased frailty becomes more pronounced with advancing age^21,22^. Physical frailty is defined as a condition characterized by weight loss, decreased vitality, reduced physical activity, slower walking pace, and diminished grip strength^22^, with musculoskeletal functioning being a dominant component. Therefore, physical frailty is highly prevalent among older adults with arthritis^23^. In 2022, a meta-analysis of 16 studies reported a prevalence of 33.5% and 39.9% for frailty and pre-frailty in patients with RA, respectively^24^. Recently, the concept of frailty has been expanded to include not only physical aspects, but also mental, social, and cognitive aspects^21^. It is especially noteworthy that patients with arthritis frequently report symptoms including pain, functional impairment, depression, and social isolation^4,25^. Hence, there is a need to employ the concept of frailty in patients with arthritis, encompassing not only physical frailty but also mental and social aspects. Cognitive impairment is a component of frailty, and the relationship between frailty and cognitive impairment has been extensively studied^26^. Although most patients with arthritis are not robust, the effects of frailty stratification on cognitive decline in patients with arthritis remain unclear.

Pain acts as a stressor and contributes to the development of frailty, with several studies indicating a significant association between pain and frailty^3,27,28^. Furthermore, pain is associated with cognitive decline^19,20^, and frailty is also linked to cognitive deterioration^26^. Notably, pain and frailty are typical characteristics of patients with arthritis. Considering the relationship among pain, frailty, and cognitive function, we hypothesized that older adults with arthritis experiencing uncontrolled pain are more likely to experience greater cognitive decline. However, to date, no studies have examined the combined effects of pain and frailty on cognitive function in older adults with arthritis. This study will assist in stratifying and identifying subgroups of people with arthritis at risk of cognitive decline, laying the foundation for fundamental research into preventing cognitive decline. Therefore, the primary objective of this study is to identify the factors affecting cognitive function and determine how severity of frailty and pain impact cognitive function in older adults with arthritis.

## Methods

All methods were carried out in accordance with relevant guidelines and regulations.

### Study design and participant selection

This cross-sectional study aimed to identify the factors affecting cognitive function and determine how frailty severity and pain affect cognitive function in older adults with arthritis, using the seventh and eighth survey data of the Korean Longitudinal Study of Aging (KLoSA). The KLoSA survey methods have been described previously^29^.

**Figure 1:**
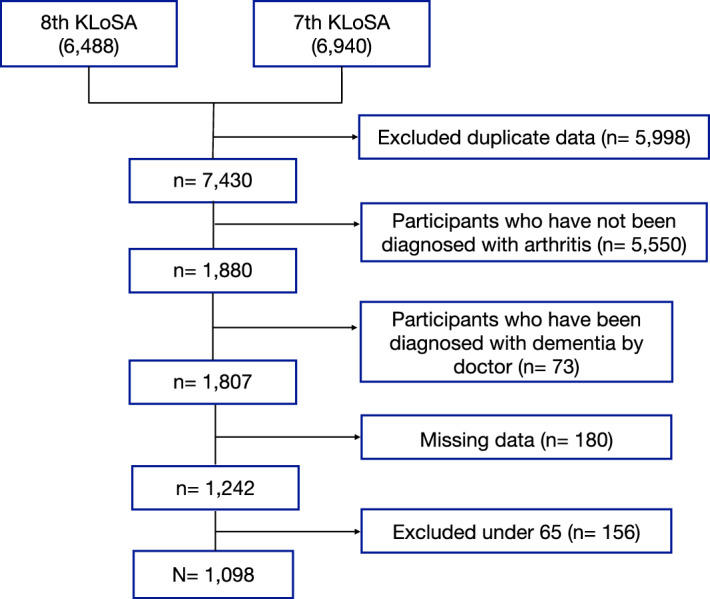
The participant-selection flow chart. *KLoSA* Korean Longitudinal Study of Aging.

The seventh and eighth surveys used in this study included 6940 and 6488 participants, respectively, and were undertaken in 2018 and 2020, respectively. Arthritis is prevalent in all age groups, but older adults have distinctive characteristics; therefore, the study was limited to patients with arthritis aged 65 years or older. Individuals who responded “yes” to the question “Have you ever been diagnosed with arthritis by a doctor?” were enrolled as participants. To maintain data consistency, when participants took part in both the seventh and eighth surveys, only the data from the eighth survey were considered. Exclusion criteria were as follows: (1) data from the seventh survey were disregarded in cases of duplicate participation in both surveys; (2) variables with missing data; (3) patients diagnosed with dementia by a physician; and (4) patients aged < 65 years. Although arthritis is a chronic disease, there are cases where the disease develops before 65 years of age; therefore, there are limitations in generalizing the results of this study to all patients with arthritis. A flowchart of the participant selection process is shown in Fig. 1.

### Measures

#### Cognitive function

The Korean version of the Mini-Mental State Examination (K-MMSE) measures cognitive function and is widely used as a screening test for patients with dementia^30^. Moreover, the K-MMSE is not only employed to identify dementia in research settings but also to gauge general cognitive function^31,32^. It consists of 19 items, each scored from 0 to 30 points, with higher scores indicating better cognitive function^30^. A K-MMSE score of 17 points or less may warrant the suspicion of dementia, while a K-MMSE score ranging from 18 to 23 points may indicate mild cognitive impairment^33^. Reliability testing for community-dwelling older adults demonstrated a Cronbach alpha of 0.89^34^ and the Cronbach alpha of this study overall was 0.73.

#### Pain

Participants were categorized according to their responses to the question, “Do you currently experience difficulties in performing daily activities due to pain?”. Those participants answering “yes” were classified as experiencing severe pain, whereas those answering “no” were classified as experiencing no severe pain.

#### Frailty

Frailty encompasses physical (weakness), psychological (exhaustion), and social (isolation) determinants^35,36^. Physical weakness was denoted by grip strength below 24 kg for men and 15 kg for women. Psychological exhaustion was indicated by the feeling of not being able to do anything at all for more than three to four days in a week. Social isolation was defined as minimal or lack of participation in social groups (alumni associations, hometown associations, etc.), religious groups (churches, cathedrals, etc.), cultural activities groups (choirs, plays, movies, etc.), sports and leisure groups (mountain clubs, etc.), civic groups, interest groups and political groups, volunteer groups, and learning groups (college or classes for older adults, etc.). Participants meeting two or more of the determinants were classified as frail; those meeting one criterion were defined as pre-frail, and those meeting none of the determinants were considered robust^35,36^.

#### Group by severe pain and frailty status

Participants were categorized into six groups according to their level of severe pain and frailty status: robust, severe pain only, only pre-frail, pre-frail with severe pain, frail only, and frail with severe pain.

#### General characteristics

General characteristics included age, sex, level of education, and marital status. Highest education levels of participants were classified as elementary school, middle school, and high school or higher. Marital status was distinguished by the presence or absence of a current spouse or common-law partner.

#### Health behavior and health condition

Smoking status was categorized as current smokers or non-smokers. Drinking status was classified as current drinkers and non-drinkers. Health conditions included chronic diseases, hearing impairments, and visual impairments. The number of chronic diseases was classified as 0, 1, 2, or 3 or more for chronic diseases diagnosed by doctors, and included hypertension, diabetes, cancer, or lung, liver, heart, cerebrovascular, psychiatric, and spinal diseases. Hearing and visual impairments were measured on a Likert scale from 1 to 5, with higher scores indicating more severe impairment, represented as continuous variables.

### Statistical analyses

The data were analyzed using R version 4.0.0 (R Core Team, 2020) with significance set at *p* < 0.05. Using descriptive statistics, the participant’s general characteristics, health behaviors, health conditions, and cognitive functions are expressed as real numbers, percentages, and means and standard deviations, depending on the characteristics of the variables. To investigate the differences in cognitive function according to categorical variables, independent t-tests and analysis of variance were used. Pearson’s correlation coefficients were calculated to investigate the relationship between cognitive function and continuous variables. The distribution of the K-MMSE scores by group is presented as a density plot (Fig. 3). For multivariate regression analysis, variables were considered statistically significant if the 95% confidence interval (CI) did not include zero.

### Ethical considerations

The Institutional Review Board of G University (approval no. GIRB-D23-NX-0027) approved this study and waived the requirement for obtaining written informed consent.

## Results

Of the 13,428 participants at baseline (6488 from the eighth survey and 6940 from the seventh survey), 1, 098 participants were selected after the exclusion of those without a confirmed arthritis diagnosis as well as those with duplicate entries (n = 5998), participants not diagnosed with arthritis (n = 5550), participants with dementia (n = 73), those with missing data (n = 180), and those under the age of 65 years (n = 156).

### Characteristics of study participants

The participants’ characteristics are presented in Table 1. Among the 1098 participants, the average age was 77.1 ± 6.8 years with approximately 79.0% females (n = 867) and 57.7% married individuals (n = 634). More than one-third of participants were elementary school graduates (n = 753, 68.6%). Most participants were non-smokers (n = 1052, 95.8%) or non-drinkers (n = 912, 83.1%). More than one-third of the patients had more than two chronic diseases (n = 494, 45%). Visual and hearing impairment scores were 3.2 ± 0.7 and 2.8 ± 0.7, respectively. In particular, 45.3% of participants experienced severe pain, 42% were robust, 37.1% were pre-frail, and 20.9% were frail. The average K-MMSE score of the participants was 22.9 ± 5.5. Moreover, 30.7% of participants were suspected to have mild cognitive impairment, and 16.9% were suspected to have dementia.

**Table 1:** Participant characteristics and MMSE scores (n = 1098).

Variable	n (%) or M ± SD
General	
Age	77.1 ± 6.8
Sex	
Female	867 (79.0)
Male	231 (21.0)
Marital status	
Not married	464 (42.3)
Married	634 (57.7)
Final education	
Elementary	753 (68.6)
Middle	175 (15.9)
High school or higher	170 (15.5)
Health behavior	
Smoking status	
Non-smoker	1052 (95.8)
Smoker	46 (4.2)
Drinking status	
Non-drinker	912 (83.1)
Drinker	186 (16.9)
Health condition	
Number of diseases	
None	242 (22.0)
One	362 (33.0)
Two or more	494 (45.0)
Visual impairment (1–5)	3.2 ± 0.7
Hearing impairment (1–5)	2.8 ± 0.7
Severe pain	
No	601 (54.7)
Yes	497 (45.3)
Frailty status	
Robust	461 (42.0)
Prefrail	407 (37.1)
Frail	230 (20.9)
MMSE score (0–30)	22.9 ± 5.5
24 ≤	575 (52.4)
18 ≤ ≤ 23	337 (30.7)
≤ 17	186 (16.9)

*M* mean, *SD* standard deviation, *MMSE* Mini-Mental State Examination.

**Figure 2:**
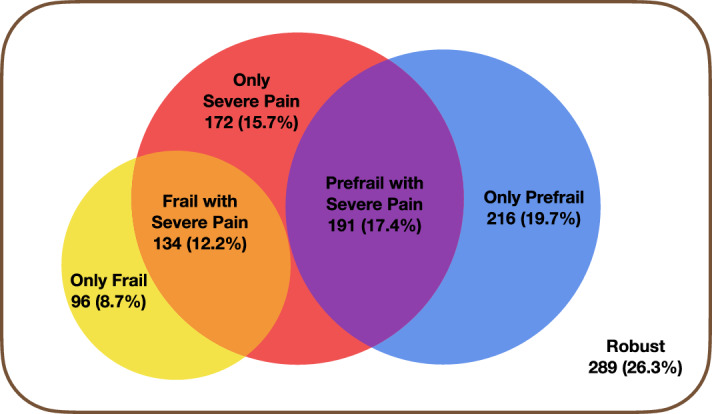
Groups categorized by severe pain and frailty status (n = 1098).

For participants categorized in groups according to the severity of pain and frailty status, 26.3% were robust, 15.7% experienced only severe pain, 19.7% were only prefrail, 17.4% were pre-frail with severe pain, 8.7% were only frail, and 12.2% were frail with severe pain (Table 2, Fig. 2).

**Table 2:** Groups categorized by pain severity and frailty status (n = 1098).

Group	n (%) or M ± SD
Robust	289 (26.3)
Only severe pain	172 (15.7)
Only prefrail	216 (19.7)
Prefrail with severe pain	191 (17.4)
Only frail	96 (8.7)
Frail with severe pain	134 (12.2)

### Differences in K-MMSE according to categorical variables

The differences between the categorical variables and K-MMSE scores are presented in Table 3. In terms of demographic characteristics, there were significant differences in the K-MMSE scores according to sex (t = − 3.7, *p* < 0.001), marital status (t = − 7.1, *p* < 0.001), final education (F = 56.6, *p* < 0.001), drinking status (t = − 6.4, *p* < 0.001), number of diseases (F = 7.1, *p* < 0.001), severe pain (t = 7.1, *p* < 0.001), and frailty status (F = 142.1, *p* < 0.001).

**Table 3:** MMSE scores according to categorical variables (n = 1098).

	M ± SD	F or t	*p*
Sex		− 3.7	< 0.001
Female	22.6 ± 5.6		
Male	24.1 ± 5.1		
Marital status		− 7.1	< 0.001
No	21.6 ± 5.8		
Yes	23.9 ± 5.1		
Final education		56.6	< 0.001
Elementary^a^	21.8 ± 5.6		^a<b,c^
Middle^b^	25.3 ± 4.3		
High school^c^	25.6 ± 4.4		
Smoking status		− 0.9	0.370
Non-smoker	22.9 ± 5.5		
Smoker	23.6 ± 5.6		
Drinking status		− 6.4	< 0.001
Non-drinker	22.6 ± 5.7		
Drinker	24.9 ± 4.2		
Number of diseases		7.1	< 0.001
None^a^	23.8 ± 5.3		^a<b^
1	23.3 ± 5.3		
2^b^	22.3 ± 5.6		
Severe pain		7.1	< 0.001
No	24.0 ± 5.0		
Yes	21.6 ± 5.8		
Frailty status		142.1	< 0.001
Robust^a^	25.3 ± 4.0		^c,b<a^
Prefrail^b^	22.6 ± 5.1		^c<b^
Frail^c^	18.8 ± 6.2		
Groups by severity of pain and frail status			< 0.001
Robust^a^	25.8 ± 3.7		^f,e,d,c<a^
Only severe pain^b^	24.6 ± 4.2		^d,e,f<b,c^
Only prefrail^c^	23.6 ± 4.5		^e<d^
Prefrail with severe pain^d^	21.5 ± 5.4		^f<e^
Only Frail^e^	19.5 ± 6.3		
Frail with severe pain^f^	18.1 ± 6.0		

*M* mean, *SD* standard deviation, *MMSE* Mini-Mental State Examination.

**Figure 3:**
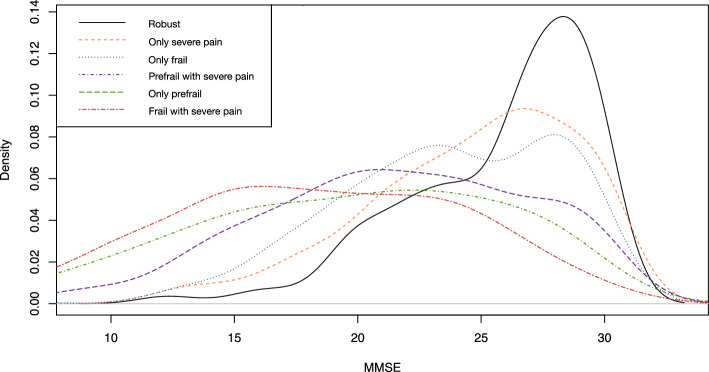
Density plot of cognitive function by severe pain and frailty status groups. *K-MMSE* Korean version of Mini-Mental Statement Examination.

In addition, K-MMSE scores varied significantly based on the presence of severe pain and frailty status (F = 64.39, *p* < 0.001). The distribution of K-MMSE scores in the robust group was positively skewed. Conversely, the frail with severe pain group showed a concentration of lower K-MMSE scores (shown in Fig. 3).

### Correlation between continuous variables and K-MMSE

The correlations between continuous variables and K-MMSE scores are presented in Table 4. There was a negative correlation between K-MMSE scores and age (*r* = − 0.41, *p* < 0.001), visual impairment (*r* = − 0.27,* p* < 0.001), and hearing impairment (*r* = − 0.32, *p* < 0.001).

**Table 4:** Relationship between MMSE scores and continuous variables (n = 1098).

Division	Age	Visual impairment	Hearing impairment
MMSE	− 0.41*	− 0.27*	− 0.32*

Spearman’s correlation coefficient was used to assess the relationship between continuous variables and MMSE scores. *MMSE* Mini-Mental State Examination. *p < 0.001.

### Multiple linear regression analysis

Multiple linear regression analysis was used to identify the factors associated with K-MMSE scores (Table 5). The model accounted for 37.1% of the variability observed in the K-MMSE scores. Advanced age was associated with a decrease in K-MMSE scores (β = − 0.16, 95% CI [− 0.21, − 0.12]), as were visual (β = − 0.95, 95% CI [− 1.37, − 0.53]) and hearing impairments (β = − 1.22, 95% CI [− 1.61, − 0.83]). Additionally, demographic factors were associated with higher K-MMSE scores, including male sex (β = 1.11, 95% CI [0.38, 1.85]), middle school education (β = 1.49, 95% CI [0.74, 2.24]), and high school or higher education (β = 1.70, 95% CI [0.92, 2.47]). Compared to the robust group, only prefrail (*β* = − 1.54, CI = − 2.33 to − 0.76), prefrail with severe pain (*β* = − 2.69, CI = − 3.52 to − 1.87), only frail (*β* = − 4.02, CI = − 5.08 to − 2.97), and frail with severe pain (*β* = − 5.03, CI = − 5.99 to − 4.08) groups were associated with lower K-MMSE scores.

**Table 5:** Multivariate linear regression for MMSE scores in older adults (n = 1098).

	*β*	s.e.	*CI*	*P*
Age	− 0.16	0.02	− 0.21; − 0.12	< 0.001
Sex				
Female	Ref (0)			
Male	1.11	0.37	0.38; 1.85	.003
Marital status				
No	Ref (0)			
Yes	0.54	0.30	− 0.04; 1.13	.067
Final education				
Elementary	Ref (0)			
Middle	1.49	0.38	0.74; 2.24	< 0.001
High school or over	1.70	0.40	0.92; 2.47	< 0.001
Drinking status				
Non-drinker	Ref (0)			
Drinker	0.42	0.38	− 0.33; 1.16	.275
Number of chronic diseases				
None	Ref (0)			
One	0.47	0.37	− 0.26; 1.19	.205
Two	0.00	0.35	− 0.69; 0.69	.994
Visual impairment	− 0.95	0.21	− 1.37; − 0.53	< 0.001
Hearing impairment	− 1.22	0.20	− 1.61; − 0.83	< 0.001
Group by severity of pain and frail status				
Robust	Ref (0)			
Only severe pain	− 0.69	0.42	− 1.51; 0.14	.104
Only prefrail	− 1.54	0.40	− 2.33; − 0.76	< 0.001
Prefrail with severe pain	− 2.69	0.42	− 3.52; − 1.87	< 0.001
Only frail	− 4.02	0.54	− 5.08; − 2.97	< 0.001
Frail with severe pain	− 5.03	0.49	− 5.99; − 4.08	< 0.001
R^2^	0.380			
Adjusted R^2^	0.371			
Residual s.e.	4.4			
*F*	44.1 (< 0.001)			

*s.e.* standard error, *CI* confidence interval, *Ref* reference, *MMSE* Mini-Mental State Examination.

## Discussion

To the best of our knowledge, this study is the first to examine the factors affecting cognitive function based on the severity of pain and frailty levels in individuals with arthritis, using a nationally representative population in Korea. The findings of this study suggest that various factors including age, sex, level of education, visual and hearing impairments, severe pain, and frailty status are correlated with cognitive function. We observed that cognitive function deteriorated as frailty progressed and severe pain occurred. In individuals experiencing only severe pain, cognitive function scores did not differ from those of the robust group. However, when prefrail and frailty was present, cognitive function scores were lower than those in the robust group. Notably, the group experiencing both frailty and severe pain had the lowest cognitive function scores, highlighting the substantial impact of frailty severity and the coexistence of severe pain with arthritis in older adults.

In terms of demographic characteristics, sex was a significant factor, with almost 80% of the participants in our study being female. This proportion was slightly higher than that reported in previous studies, wherein female participants accounted for approximately 66–78% of the total participants^37,38^. One possible explanation for this discrepancy is that previous studies focused solely on RA or OA, whereas our study encompassed both types of arthritis and exclusively targeted older adults. Globally, RA and OA are more prevalent in women^3,39^. For women who experience hormonal changes, such as pregnancy, childbirth, or menopause, the number of pregnancies is negatively associated with the onset of RA^40^. Conversely, as estrogen levels decrease after childbirth, the risk of developing RA increases^41^. Moreover, women undergoing menopause experience a rapid decline in estrogen levels, which is associated with an increased risk of developing RA^42^. Similar to RA, it has been established that estrogen also plays a crucial role in OA. Given that estrogen may confer protective effects against cartilage damage, osteophytosis, and alterations in the subchondral bone of the joints^43^, the notably higher proportion of women in this study could be attributed to these factors. Given the distinct female-specific characteristics associated with arthritis, there is a potential limitation in generalizing our findings to both sexes. Therefore, for future investigations, it is advisable to delve into a sex-specific analysis to comprehensively examine the factors influencing the condition.

The present study denoted approximately 31% of the participants as frail and 37% as prefrail. According to a recent meta-analysis of patients with RA, the prevalence of frailty was 33.5% and that of prefrailty was 24.7%^24^. In contrast, a previous study conducted in older adults with OA reported that the prevalence of frailty and prefrailty was 12.9% and 65.6%, respectively^38^. The prefrailty and frailty incidence rates differed depending on RA and OA: the RA group was found to have a higher frailty incidence rate, while the OA group had a higher prefrailty incidence rate. This discrepancy in occurrence rates is supported by Cook’s analysis of big data^44^, which revealed frailty ratios of 2.8 (95% CI 1.7, 4.6) in the RA group and 1.7 (95% CI 1.3, 2.1) in the OA group. The present study, which included both groups, suggests a moderate occurrence of frailty and prefrailty. Despite variations in the reported prevalence rates of frailty and prefrailty among patients with arthritis in different studies due to diverse frailty assessment tools, a consistent observation emerged: frailty and prefrailty are prevalent among patients with arthritis, encompassing individuals with RA and OA.

Herein, factors influencing cognitive function in patients with arthritis included older age, female sex, lower educational levels, and visual or hearing impairment. Older age, female sex, and lower educational level are well-known factors associated with cognitive impairment^45^. In addition, visual and hearing impairments were negatively correlated with cognitive function, supporting the results of previous studies^46–48^. More specifically, dual impairment in visual and hearing acuity accelerates the rapid cognitive decline^49^. Functional limitations, such as impaired visual or hearing, are modifiable factors. Healthcare providers should assess the patients' conditions and offer appropriate assistance, including eyeglasses or hearing aids. Consequently, when identifying the high-risk group for cognitive impairment among patients with arthritis, it is imperative to consider not only arthritis-related factors but also educational levels and sensory impairments in the screening process.

According to previous research, moderate alcohol consumption among older adults for social purposes may contribute to higher cognitive function compared to that of non-drinkers. This is because they engage in social activities that expose them to stimuli and interpersonal relationships, which may alleviate feelings of loneliness that negatively impacts cognitive function^50,51^. Conversely, excessive alcohol consumption has been associated with detrimental health conditions, such as cognitive decline, contributing to a decrease in brain activity^52^. However, status of alcohol consumption was not significant in this study. A plausible explanation for this finding is that the current study focused on older adults diagnosed with arthritis. In addition, the study consisted of predominantly female participants, with hormonal influences potentially leading older women to have a significantly lower rate of alcohol consumption than men^53^. The sex-based disparity in this study may account for the lack of significant findings regarding alcohol consumption. Therefore, subsequent research involving subgroup analyses should be conducted to examine the effects of drinking status in older adults with arthritis, considering sex-related differences in cognitive function based on alcohol consumption status.

When analyzing groups based on the presence of severe pain and frailty, severe pain alone did not have a substantial impact on cognitive function. A key finding revealed that participants with severe pain maintained their K-MMSE scores within the normal range of intact cognitive function, in contrast to those with frailty who fell within the range of mild cognitive impairment. Furthermore, when participants experienced frailty and severe pain concurrently, the synergistic effect led to a more pronounced decline in cognitive function. In other words, the group with both frailty and severe pain exhibited lower mild cognitive impairment scores, indicating a more significant negative impact on cognitive function than either factor alone. Pain is a well-known symptom of arthritis^54,55^, and its significant impacts on cognitive dysfunction were confirmed by previous studies^56,57^. Frailty is also strongly associated with cognitive function, as explained by the accumulation of deficits model^58^ and the phenotype-based model^22^. Severe pain can be construed as a factor contributing to activity limitations^59^ that expedites the progression of physical frailty. Furthermore, it may instigate frailty in mental and social aspects, thereby resulting in a multistep sequence of depressive symptoms, pain progression, and functional limitation, which further reduces cognitive function. However, because this study is a cross-sectional study, it is difficult to provide a sufficient explanation of the potential mechanism. Therefore, in future research, a longitudinal study is needed to determine how pain, frailty, and their combined effects affect cognitive function in patients with arthritis. To the best of our knowledge, this study is the first attempt to identify prominent risk factors among the prevailing symptoms in older adults with arthritis through group analysis. It has been established that frailty takes precedence over severe pain in terms of its impact. Consequently, when healthcare providers screen and devise intervention strategies for cognitive function, they should consider the level of frailty. Furthermore, for older adults with arthritis experiencing both frailty and severe pain, it is essential to acknowledge that their cognitive function can deteriorate to levels resembling dementia. This awareness is crucial for the timely screening for high-risk dementia and for personalized care tailored to the individual’s physical and cognitive needs.

This study had some limitations. First, owing to the nature of the secondary data, our study included patients with arthritis as a whole. Questionnaires based on the original data were designed to confirm the diagnosis of arthritis without distinguishing between RA and OA. To gain insight into the factors influencing disease characteristics, the OA and RA groups should be separated and analyzed. Second, the detailed categorization of pain severity and incorporation of pain site variables, which are vital components of comprehensive pain assessment, were restricted due to the inherent limitations in the analysis of secondary data. Third, the stringent exclusion criteria applied to the study participants impose constraints on the generalizability of the research findings. In the context of RA, the onset of the disease frequently occurs prior to the age of 65, and a notable portion of patients with arthritis includes patients with dementia. Finally, while these findings may highlight correlations or associations between variables, they did not establish direct causation or influence among pain, frailty, or cognitive function. Therefore, a longitudinal study is needed to determine whether the combined effect of frailty and pain further reduces cognitive function.

## Conclusion

This was a cross-sectional descriptive study that involved secondary data analysis. We identified the relationship among pain, frailty, and cognitive function and provided fundamental data for the development of an intervention program aimed at improving pain, frailty, and cognitive function in older adults with arthritis. In this study, the severity of frailty and severe pain in older adults with arthritis influenced cognitive decline. It is noteworthy that the simultaneous occurrence of frailty and severe pain resulted in a synergistic impact, exacerbating the decline in cognitive function to a greater extent. In the clinical setting, healthcare providers should focus on the regular assessment and timely detection of cognitive impairment for the application of interventions to improve cognitive function according to general characteristics, frailty severity, and pain in older adults with arthritis. Additionally, as this study adopted a cross-sectional design, it could not validate the causal connection between pain, frailty, and cognitive function. To elucidate the mechanism behind the decline in cognitive function among patients with arthritis and the combined effect of pain and frailty, it is imperative to accurately delineate the relationships through subsequent longitudinal studies.

## Data Availability

The data supporting the results of this research can be accessed through the Korean Longitudinal Study of Aging website at http://survey.keis.or.kr.
